# Thermosensitive core-rigid micelles of monomethoxy poly(ethylene glycol)-deoxy cholic acid

**DOI:** 10.1186/s40824-022-00263-9

**Published:** 2022-04-28

**Authors:** Jin Ok Han, Hyun Jung Lee, Byeongmoon Jeong

**Affiliations:** Department of Chemistry and Nanoscience, Ewha Womans University, 52 Ewhayeodae-gil, Seodaemun-gu, Seoul, Korea

**Keywords:** Deoxy cholic acid, Micelle, Core rigidity, Thermosensitivity

## Abstract

**Background:**

Thermosensitive micelles with rigid cores that exhibit a reversible lower critical solution temperature at 30–35 °C can be applied for drug delivery.

**Method:**

Hydrophilic monomethoxy poly(ethylene glycol) was conjugated to hydrophobic deoxycholic acid to prepare monomethoxy poly(ethylene glycol)-deoxycholic acid (mPEG-DC). Micelle formation and thermosensitive solution behavior were studied using various methods, including hydrophobic dye solubilization, transmission electron microscopy, dynamic light scattering, turbidity measurement, microcalorimetry, and ^1^H-NMR spectroscopy. Drug release from the thermosensitive micelles was demonstrated using estradiol, a model drug.

**Results:**

The mPEG-DC formed micelles with a critical micelle concentration of 0.05 wt.% and an average size of 15 nm. Aqueous mPEG-DC solutions exhibit a lower critical solution temperature (LCST) that is independent of concentration and reversible over heating and cooling cycles. The LCST transition is an entropically driven process involving dehydration of the PEG shell. The thermosensitive mPEG-DC micelles with rigid DC cores were applied as an estradiol delivery system in which estradiol was released, without initial burst, over the 16 days in a diffusion-controlled manner.

**Conclusions:**

This study suggests that mPEG-DCs form thermosensitive micelles with rigid cores that can function as an excellent diffusion-controlled hydrophobic drug delivery system without initial burst release.

**Graphical Abstract:**

Thermosensitive core-rigid micelles of monomethoxy poly(ethylene glycol)-deoxy cholic acid
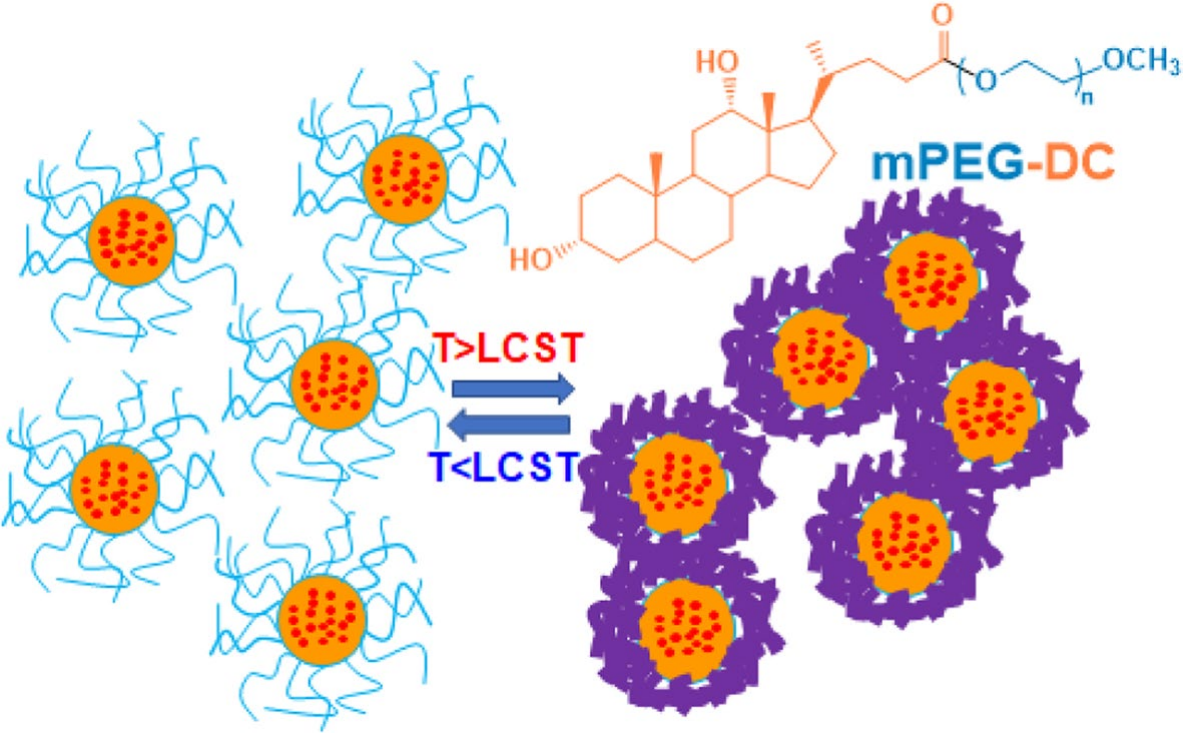

**Supplementary Information:**

The online version contains supplementary material available at 10.1186/s40824-022-00263-9.

## Background

Thermosensitive micelles have been extensively investigated as carriers in drug delivery systems [1, 2]. The thermosensitive polymers are hydrated and have extended conformations below their lower critical solution temperature (LCST). In contrast, they are dehydrated and collapsed above their LCST. Poly(N- isopropylacrylamide) (PNIPAAm), poly(2-isopropyl-2-oxazoline), poly(2-(2-ethoxy)ethoxy ethyl vinylether), poly(poly(ethylene glycol) methylether methacrylate) (polyPEGMA), and poly(N-vinyl caprolactam) are typical examples of thermosensitive polymers used in thermosensitive micelles with LCST close to body temperature of 37 °C [3–8]. Block copolymers consisting of a hydrophobic block and a hydrophilic block are the most common examples of temperature-sensitive micelles. N-(2-hydroxy propyl) methacrylamide (HPMAm) and N-(2-hydroxy propyl) methacrylamide dilactate (HPMAm-Lac2) block copolymers exhibit thermosensitivity in water as the molecular weight of hydrophobic poly(HPMAm-Lac2) increased to 15 kDa with a hydrophilic block of poly(HPMAm) with 7 kDa [9]. As the hydrophobic n-butylacrylate content increases in PNIPAM-block-poly(n-butylacrylate-co-N,N-dimethylacrylamide), the LCST decreases, and the resulting micelles become compact [10]. N-acryloyl-Ala-methylester (NAAMe) and N-acryloyl-βAla-methylester (NAβAMe) block copolymer poly(NAAMe_48_-b-NAβAMe_m_) copolymers form thermosensitive micelles with LCST which increases from 27 to 40 °C as the value of m increases from 48 to 122. Even though the two monomers are isomers, NAβAMe increases the hydrophilicity of the block polymer [11].

The core rigidity of micelles contributes to the core stability of the micelles, which provides reproducibility of drug encapsulation and release from micelles. In addition, the precise thermosensitivity and strict reversibility of the thermal transition can be related to core rigidity of the micelles [12]. A rigid-core nucleobase-thermosensitive micelle was reported using poly(propylene glycol) (M.W. = 800 Da) segments as the thermosensitive element and hydrogen-bonded uracil as a photosensitive moiety [13]. The corresponding aqueous polymer solution (5 mg/ml) exhibited an LCST of 36 − 42 °C. The uracil moiety can be cross-linked with UV light (254 nm) to form rigid-core thermosensitive micelles. Another strategy to form rigid-core micelles uses a copolymer of poly(ethylene glycol) methacrylate and D or L-poly(lactic acid)-poly(ethylene glycol) to form thermosensitive polymeric micelles [14]. By mixing L-poly(lactic acid)- and D-poly(lactic acid)-containing copolymers, a stereocomplex was formed in the micelle core, forming a rigid core. In particular, a 50/50 stereocomplex exhibits the lowest LCST compared with other compositions or enantiomeric micelles in the study.

Micelles prepared from PEG-conjugated small molecules have been mainly investigated to improve the solubility of the small molecular drug; mPEG-taxol, mPEG-docetaxel, and mPEG-doxorubicin are typical examples [15, 16]. In addition, mPEG-deoxycholic acid were also reported as micellar systems [17]. However, the molecular weight of mPEG used in the above studies was higher than 1000 Da; hence, the thermosensitive transition of the micelles in the physiologically important range of 30 − 40 °C has not been reported.

**Fig. 1:**
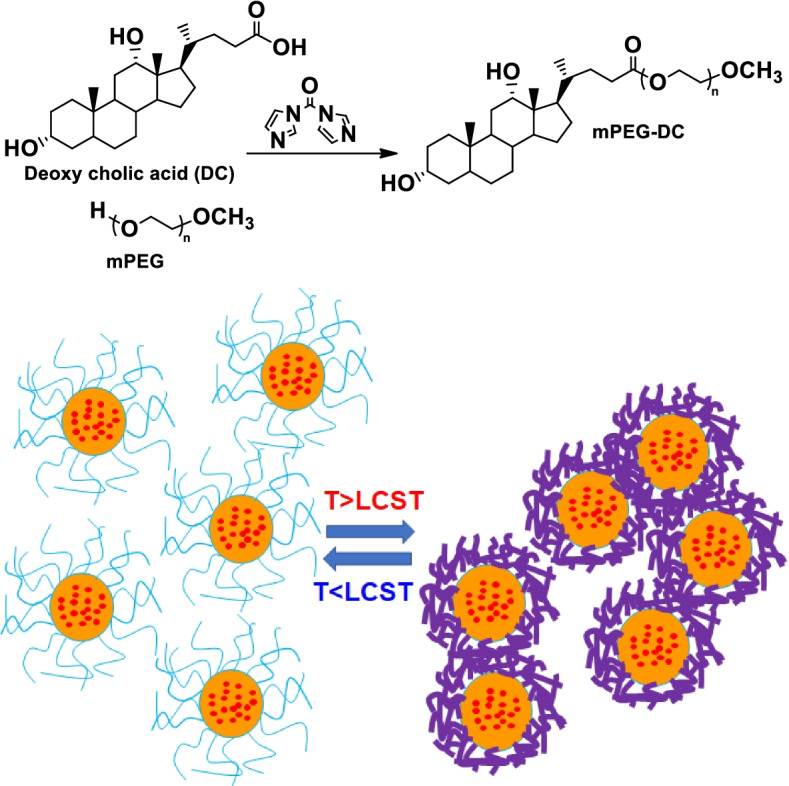
Scheme of mPEG-DC synthesis and drug release from its thermosensitive micelles. Above the LCST, mPEG shells (blue curves at below LCST/thick violet curves at above LCST) coat the micelles with rigid cores (orange circles), minimizing the initial burst release of the encapsulated drugs (red dots)

Here, we report deoxycholic acid-conjugated monomethoxy poly(ethylene glycol) (mPEG-DC) forming thermosensitive micelles with rigid cores. Deoxycholic acid (DC) is a kind of bile acid in the human body and contains two hydroxy groups on a steroid ring. Both mPEG and bile acid are biocompatible compounds. We hypothesized that thermosensitive micelles with a rigid core could minimize the initial burst release of encapsulated drugs by coating the shell at a temperature above its LCST through the thermal transition (Fig. 1).

## Methods

### Materials

Monomethoxy poly(ethylene glycol) (mPEG) (MW = 350, 550, and 750 Da), deoxycholic acid (DC), estradiol, and 1,1′-carbonyldiimidazole were used as received from Aldrich. Toluene (J.T. Baker) and tetrahydrofuran (Sigma Aldrich) were distilled over sodium before use.

### Synthesis of mPEG-DC

To prepare mPEG350-DC, mPEG (9.80 g, 28.0 mmol; MW 350 Da) was dissolved in toluene (70 ml), and the residual water was removed via azeotropic distillation at 130 °C to a final volume of 20 ml, then the flask was cooled down to 60 °C. Subsequently, 1,1′-carbonyldiimidazole (1.36 g, 8.39 mmol), DC (2.74 g, 7.0 mmol), and tetrahydrofuran (20 ml) were added to the flask, and the contents were stirred at 60 °C for 48 h. The residual solvent was removed under vacuum. The reaction mixtures were redissolved in water, and unreacted mPEG was removed under heating (precipitation)/cooling (clear solution) cycles of the solution based on its LCST. Finally, the polymers were dialyzed in water using Spectra/Por7 membranes (Spectrum Lab) with a cut-off molecular weight of 1000 Da, and then the aqueous solution was freeze dried. The yield was 55%.

### ^1^H-NMR spectroscopy

^1^H-NMR spectra of the polymer in CDCl_3_ (300 MHz NMR spectrometer, Bruker, USA) were used to determine the structure of the mPEG-DC. ^1^H-NMR spectra of the mPEG-DC in D_2_O were compared to confirm the core-shell structure of the polymer in water.

### Critical micelle concentration

The critical micelle concentration (CMC) of mPEG-DC was determined using hydrophobic dye (1,6-diphenyl-1,3,5-hexatriene) solubilization [18, 19]. The dye solution in methanol (10.0 μL at 0.4 mM) was added to an aqueous polymer solution (1.0 ml) in the concentration range of 0.0001 − 0.5 wt.%. The UV-Vis spectra of these solutions were recorded using UV-vis spectrophometer (Scinco S-3100, Korea) between 300 and 400 nm at room temperature. The intersection point of the two extrapolated lines was defined as the CMC of the mPEG-DC.

### Transmission electron microscopy

The aqueous mPEG-DC (1.0 wt.%) solution was loaded onto a copper grid, and excess water was blotted by filter paper. After air-drying at 20 °C, microscopic images of the polymer self-assembly were obtained using a transmission electron microscope (TEM; JSM-6700F, JEOL, Japan) at an accelerating voltage of 10 kV. Phosphotungstanate was used as a staining agent.

### Dynamic light scattering

The apparent size of the polymer or polymer aggregates in water (1.0 wt.%) as a function of temperature was studied using dynamic light scattering (ALV 5000-60-0). A YAG DPSS-200 laser (Langen, Germany) operating at 532 nm was used as a light source. Measurements of the scattered light were performed at an angle 90^o^ to the incident beam. The results of dynamic light scattering experiments were analyzed using the regularized CONTIN method, which is employed to analyze the autocorrelation function through inverse Laplace transform.

### LCST measurement

The absorbance at 500 nm of aqueous polymer solutions at a given concentration were measured using UV-vis spectrophotometer (Scinco S-3100, Korea) as a function of temperature from 10 to 40 °C. The solution temperature was equilibrated for 20 min at each temperature. In addition, the turbidity was monitored during heating and cooling cycles to confirm the reversibility of the phase transition.

### Microcalorimetry

A differential scanning calorimeter (VP- DSC, Microcal, USA) was used to study the heat exchange of the aqueous polymer solution (0.5 ml) at a given concentration in the temperature range of 10 − 80 °C with a heating rate of 1.0 °C/min. In addition, the thermogram was monitored during heating and cooling cycles to confirm the reversibility of the phase transition.

### Sustained release of estradiol from thermosensitive micelles

To define the solubility of estradiol in the release medium, estradiol (2.0 mg) was dissolved in phosphate-buffered saline (pH = 7.4; 1.0 ml) containing Tween 80 at concentrations of 0.0 − 5.0 wt.%. The mixture was stirred for 24 h at room temperature, and the dissolved estradiol was analyzed using a high-performance liquid chromatography (HPLC) system (Waters 1525B, USA) with a photodiode detector (Waters 2998, USA) at a wavelength of 210 nm. A Jupiter 5 μm C18 300A LC column (Phenomenex, USA) was used. Acetonitrile/water (60/40 v/v) containing trifluoroactic acid (0.1 wt.%) was used as an eluting solvent at a flow rate of 1.0 ml/min.

To prepare estradiol-loaded micelles, estradiol (3.0 mg/ml) was added to 1.0 ml of mPEG350-DC solution (20 wt.%) and stirred at 4 °C for 24 h; the solution was sonicated for 30 min at room temperature, and then centrifuged at 4000 *g* for 15 min to remove the insoluble components, if any [15]. In each formulation, the final polymer concentration was fixed at 20 wt.%.

After complete dissolution of estradiol loaded mPEG350-DC micelles in water (20 wt.% polymer in water; 1.0 ml), the solution was heated to 37 °C to form a turbid suspension. The suspension was injected into a dialysis bag (cut-off molecular weight of 3500 Da) in a vial filled with the release medium (8.0 ml) at 37 °C. The release medium was phosphate-buffered saline (pH = 7.4; 150 mM) containing 4.0 wt.% Tween 80 and sodium azide (0.02 wt.%), which allows a sink condition of the estradiol. All the release medium at 37 °C was replaced at sampling time interval. The vial was shaken at a rate of 100 strokes per minute. The released amount of estradiol was analyzed by HPLC using the method described above.

## Results

**Fig. 2:**
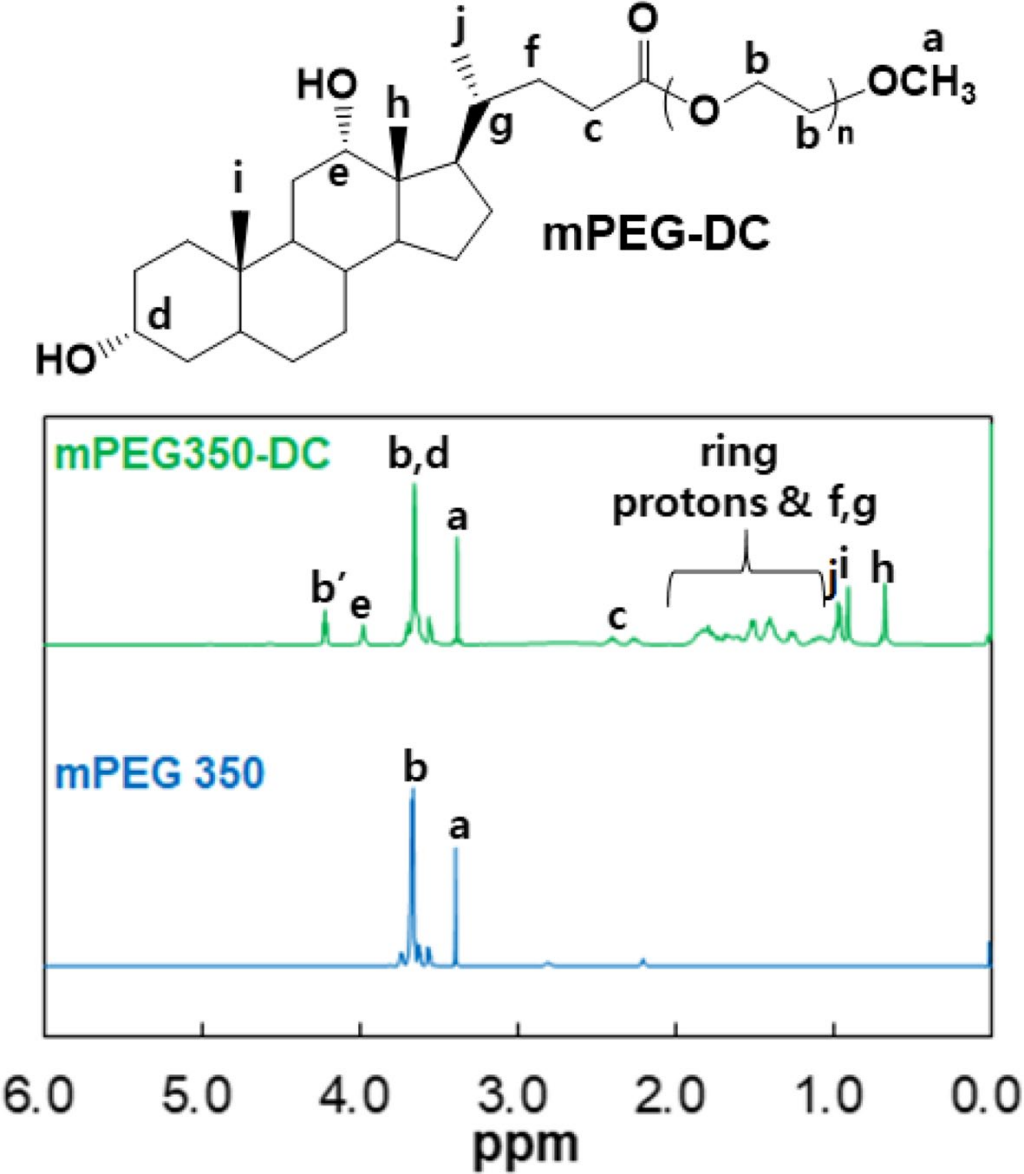
^1^H-NMR spectra of mPEG and mPEG-DC. b’ denotes the methylene peak of PEG connected to the carbonyl group

The mPEG-DC was synthesized via condensation reaction between mPEG and DC using 1,1′-carbonyldiimidazole as a catalyst. Excess mPEG was removed based on the LCST of the aqueous polymer solutions. Above their LCST, mPEG-DCs precipitate, while mPEGs remain in solution. Redissolving the polymer at low temperature followed by heating separates the mPEG-DC and removes the unreacted mPEG. Then, the mPEG-DCs were purified further using dialysis, during which the micelles remain in the membrane, while mPEG diffuses out of the membrane. ^1^H-NMR spectra of mPEG and mPEG-DC are shown in Fig. 2. The 1:1 ratio between the methyl (C18 or h in Fig. 2) peak of DC at 0.61 − 0.72 ppm and the methoxy peak of mPEG at 3.33 − 3.43 ppm confirmed the formation of mPEG-DC in a 1:1 ratio. The new triplet at 4.18 − 4.25 ppm comes from the methylene group (−COO***CH***_***2***_CH_2_-) of mPEG connected to DC, indicating the formation of mPEG-DC. All the peaks of DC at 0.61 − 2.50 ppm is related to the alkyl groups of DC [20].

**Fig. 3:**
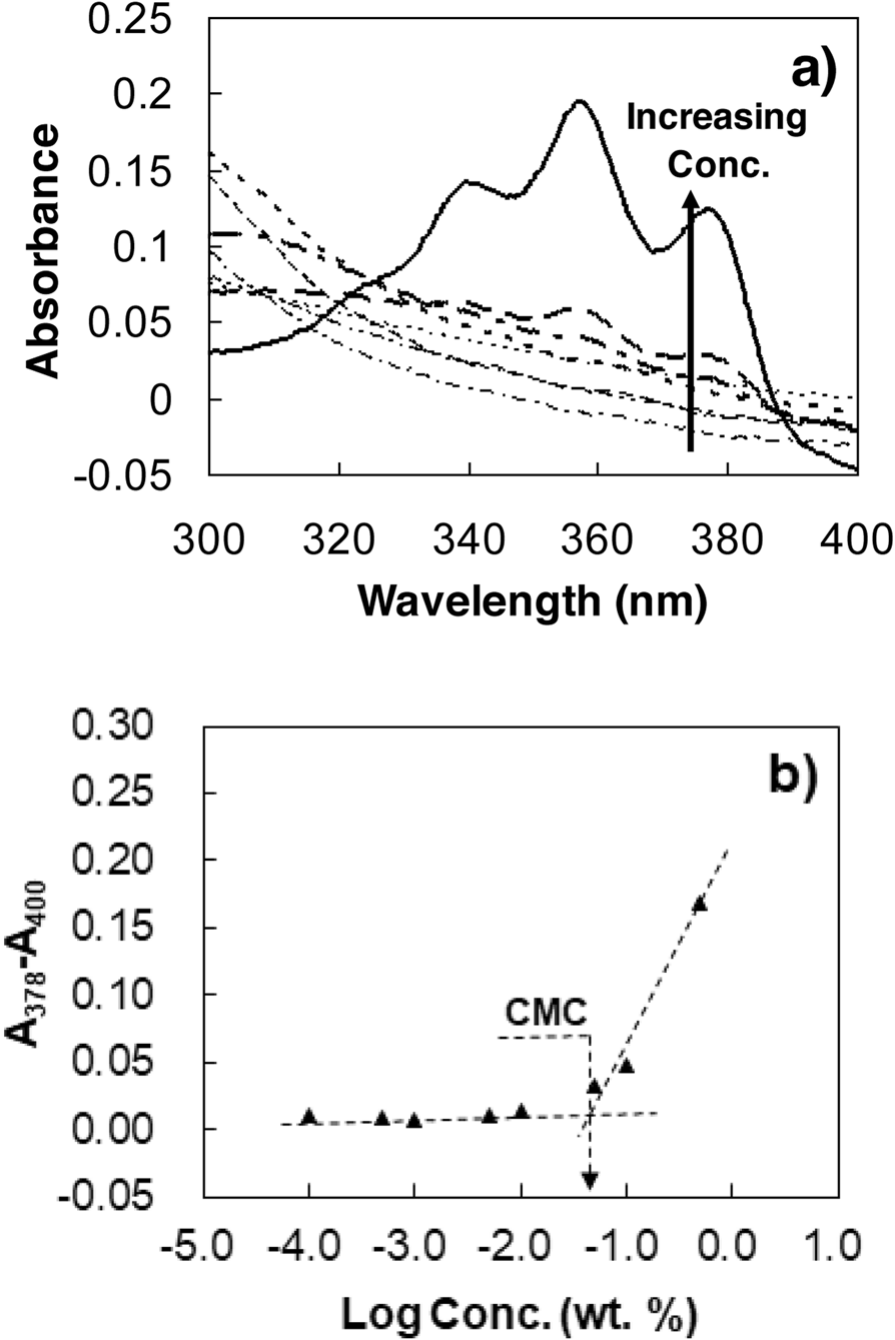
**a** Absorbance of 1,6-diphenyl-1,3,5-hexatriene in aqueous mPEG350-DC solutions. The mPEG-DC concentration was varied (0.0001, 0.0005, 0.001, 0.005, 0.01, 0.05, 0.1, and 0.5 wt.%), while the dye concentration was fixed (4.0 μM). **b** CMC determined using the intersection point of the two extrapolated lines is 0.05 wt.%

The mPEG-DC, consisting of hydrophilic mPEG and hydrophobic rigid steroid rings, self-assembles into micelles. Micelle formation was investigated using a hydrophobic dye (1,6-diphenyl-1,3,5-hexatriene). As the concentration of mPEG-DC increased, the triplet bands at 340, 360, 379 nm began to increase (Fig. 3a). The dye has a low absorption coefficient in water, but its absorption coefficient increases in a hydrophobic environment. Therefore, the increase in triplet band absorption was used to confirm micelle formation [18, 19]. The CMC of mPEG-DC, determined using the intersection point of the two extrapolated lines, is 0.05 wt.% (Fig. 3b).

**Fig. 4:**
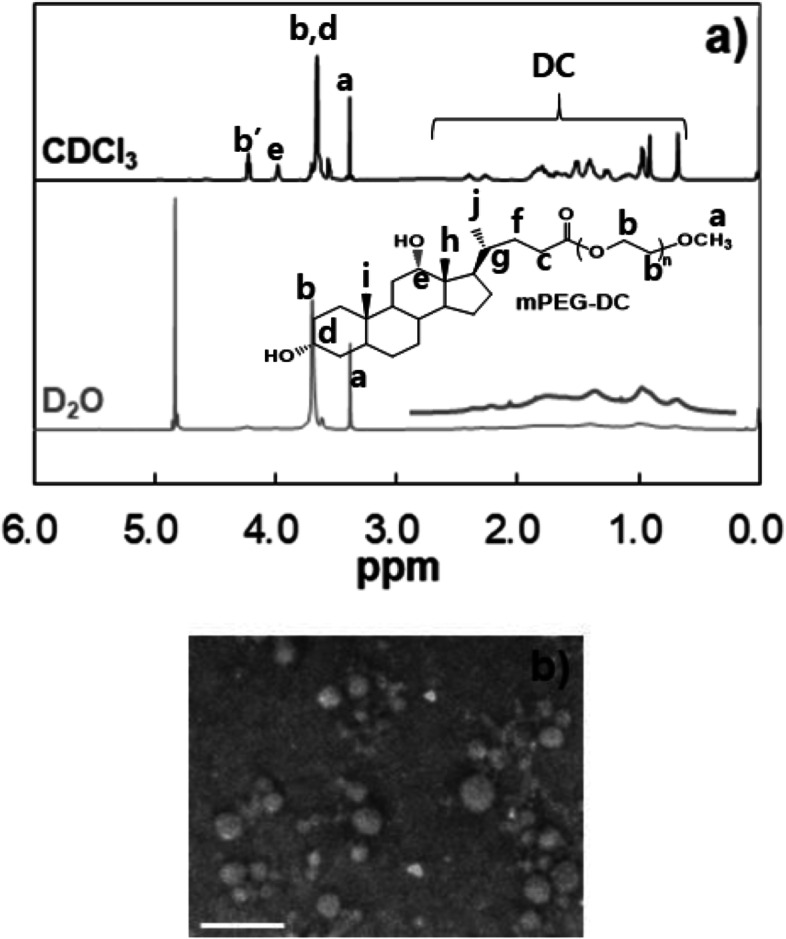
**a**^1^H-NMR spectra of mPEG350-DC in CDCl_3_ and D_2_O at 15 °C. Compared with the PEG peaks at 3.0–4.5 ppm, the DC peaks at 0.5–2.5 ppm are collapsed in D_2_O. **b** TEM images of mPEG350-DC developed from aqueous solution (1.0 wt.%). The scale bar is 50 nm

The core-shell structure of micelles was confirmed by comparing ^1^H-NMR spectra of mPEG-DC in chloroform (CDCl_3_) and in water (D_2_O). Chloroform is a good solvent for both mPEG and DC, whereas water is a good solvent for mPEG and a poor solvent for DC. Both mPEG peaks at 3.0–4.5 ppm and DC peaks at 0.5–2.5 ppm are sharp in CDCl_3_, whereas the DC peaks are collapsed in D_2_O, indicating the core (mPEG)-shell (DC) structure of mPEG-DC in water (Fig. 4a) [21]. In addition, TEM images of the mPEG-DC developed in water confirm a spherical micelle structure (Fig. 4b). Even though some deformation might occur in the self-assembly of mPEG-DC during air drying, the spherical micelle structures could be clearly seen in the TEM images. The average size of the micelles measured from the TEM images was 12 ± 5 nm, which is comparable with the results obtained from dynamic light scattering, as will be discussed next section.

**Fig. 5:**
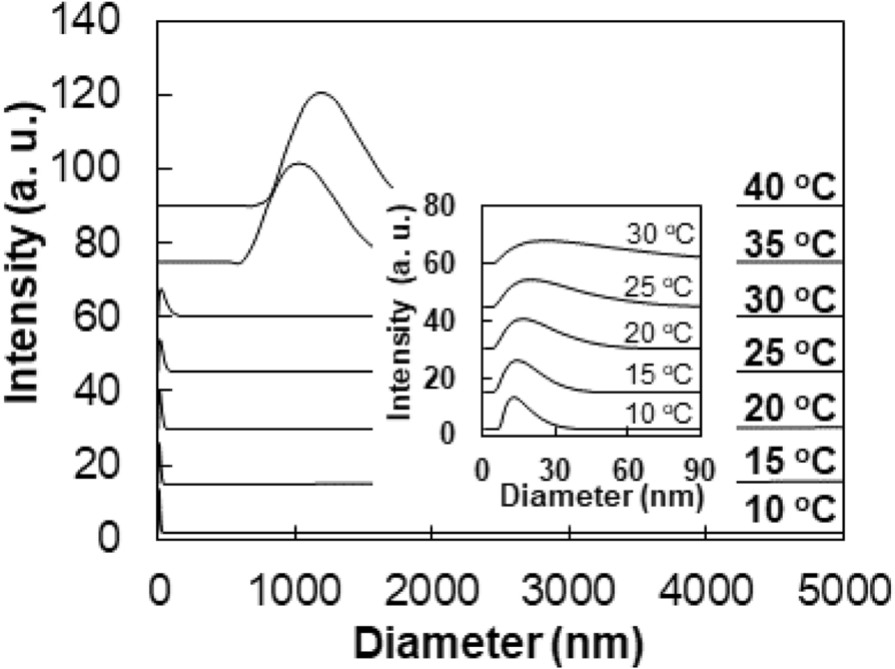
Dynamic light scattering of the mPEG350-DC aqueous solutions (1.0 wt.%) as a function of temperature. The small (0–90 nm) region is enlarged in the inset

The apparent size of the self-assembled mPEG-DC micelles was investigated as a function of temperature using dynamic light scattering (Fig. 5). The peak average size (d_p_) of micelles is 15 nm at 10 °C. Based on the hard sphere model, the aggregation number per micelle can be calculated by the following equation [22].

$$\mathrm{M}=\left({\mathrm{N}}_{\mathrm{A}}/{10}^{24}\right)\left(8{\mathrm{r}}^3/{2}^{1/2}\right)=3.43\mathrm{c}{\mathrm{r}}^3$$

M, c and r are the molar micelle mass, polymer concentration (g dm^− 3^), and radius (nm), respectively. N_A_ is the Avogadro’s number. From the polymer concentration (1.0 wt%) and the average radius (7.5 nm) of a micelle from the dynamic light scattering study at 10 °C, M is calculated to be 14,470 g mol^− 1^. Therefore, the aggregation number per micelle that is the molar micelle mass divided by the molar mass of mPEG-DC (M.W. ∼ 724 Da) is 20. The size distribution increased as the temperature increased to 15, 20, 25, and to 30 °C. As the temperature increased further to 35 and 40 °C, the particle size abruptly increased to 1110 and 1280 nm, respectively, indicating aggregation of the micelles that corresponds to the LCST of the aqueous polymer solution.

**Fig. 6:**
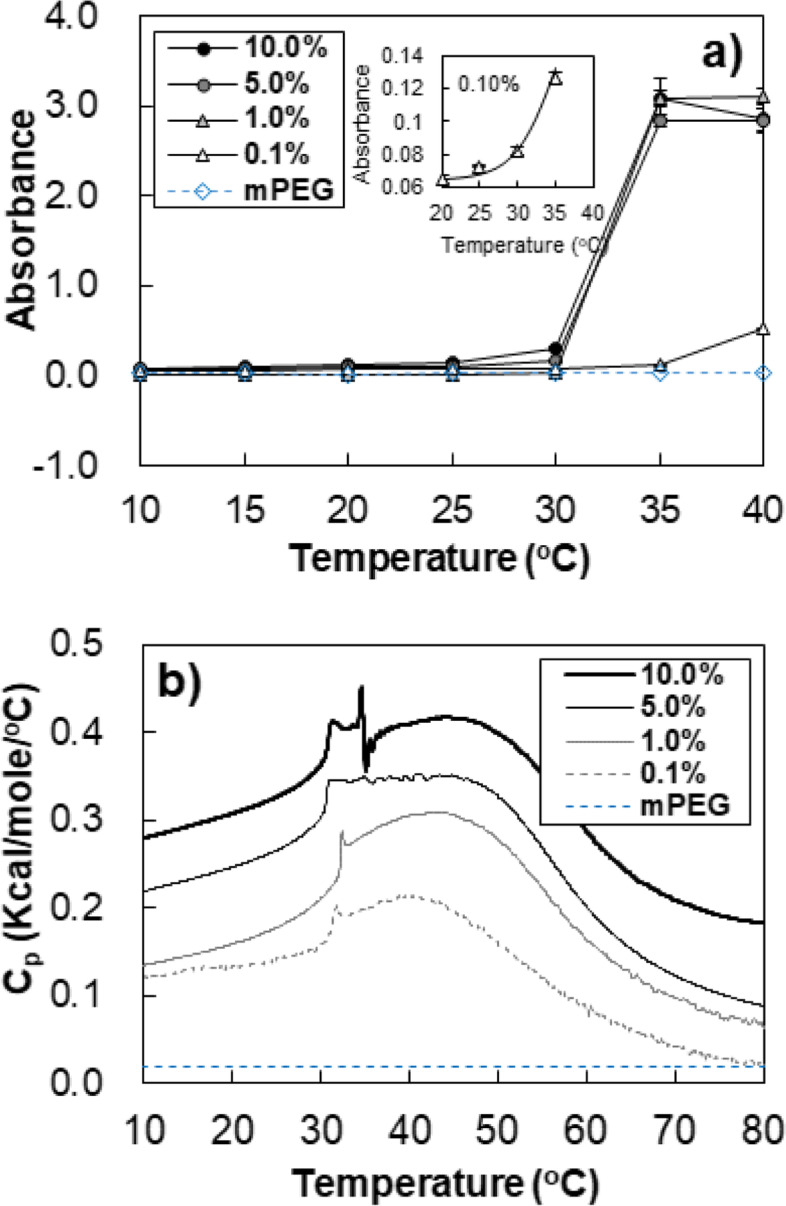
**a** Absorbance of aqueous mPEG 350-DC solutions at 500 nm as a function of temperature and concentration. An aqueous mPEG (10.0 wt.%) solution which does not exhibit LCST was used as a reference. *n* = 3. **b** Microcalorimeter thermogram of aqueous mPEG 350-DC solution as a function concentration. The concentration was varied from 0.1 to 10.0 wt.%. A thermogram of mPEG (10.0 wt.%) was used as a reference

The LCST of the aqueous mPEG-DC solution was investigated via turbidity measurement at 500 nm. Even though the polymer concentration was varied (0.1, 1.0, 5.0, and 10.0), the LCST values of the solutions were similar, 30 ~ 35 °C (Fig. 6a). The LCSTs of most thermosensitive polymers exhibit concentration-dependent behavior [22–24]. The rigid core micelles tend to exhibit a coherent transition [12].

Heat exchange during the LCST transition could be monitored using a microcalorimeter. The spike peak at 30–35 °C, followed by a large endothermic peak centered at 40–45 °C are characteristics of the transition (Fig. 6b). LCST measured by turbidity coincided with the sharp peak. The endothermic peak of the LCST indicates that the transition is caused by an entropy-driven process accompanied by dehydration of PEG or liberation of adsorbed water around the PEG. The enthalpy of the transition is proportional to the polymer concentration.

**Fig. 7:**
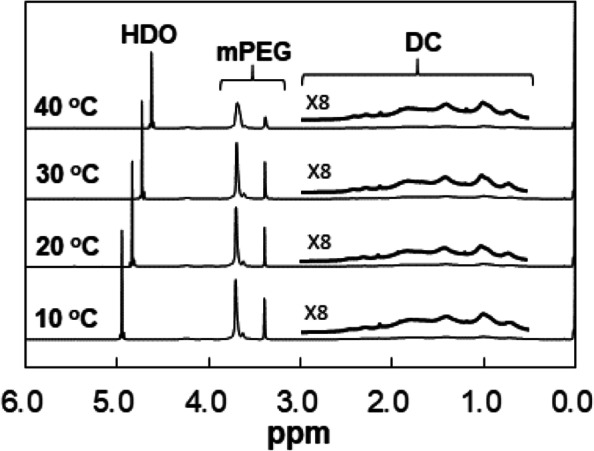
^1^H-NMR spectra of mPEG-DC (10.0 wt.% in D_2_O) as a function of temperature. Enlarged views of the DC peaks in the 0.5–3.0 ppm window are also shown

The ^1^H-NMR spectra of mPEG-DC (10.0 wt.% in D_2_O) were investigated as a function of temperature (Fig. 7). The mPEG peaks at 3.0 − 4.0 ppm collapsed at 40 °C, whereas there was no significant change in the DC peaks at 0.5 − 2.5 ppm. The collapse of the mPEG peaks indicates dehydration of the mPEG shell [25–27]. Previous thermosensitive micelles of poly(ethylene glycol)/poly(lactic acid-co-glycolic acid) (PEG/PLGA), poly(ethylene glycol)/poly(trimethylene carbonate) (PEG/PTMC), poly(ethylene glycol)/poly(propylene glycol) (PEG/PPG), and phosphoryl choline/ poly(propylene glycol) (PC/PPG) systems have hydrophobic flexible cores of PLGA, PTMC, and PPG, respectively, and hydrophilic PEG or PC shells. The NMR peak intensity of the flexible core increases as the temperature increases, while the shell peaks of PEG collapse [25–30]. The lack of increases in DC peak intensity in the ^1^H-NMR spectra reflects the rigidity of the DC core of the mPEG-DC micelles.

**Fig. 8:**
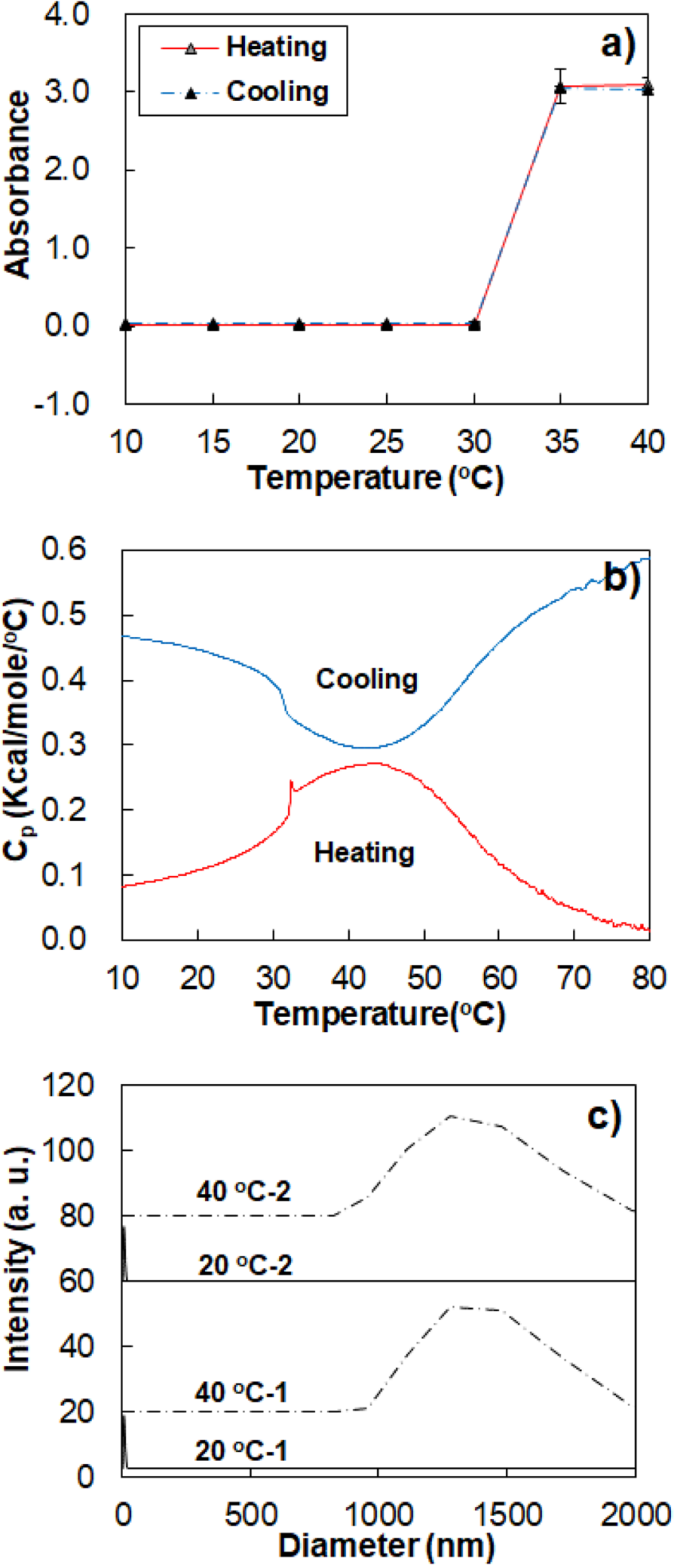
Reversibility of the thermosensitive transition of aqueous mPEG350-DC solution (1.0 wt.%). **a** The absorbance at 500 nm, *n* = 3, and **b** microcalorimeter thermograms over the heating and cooling cycles. **c** The size of the micelles at 20 °C and 40 °C. 1 and 2 indicate the first and second heating cycles, respectively

The reversibility of the thermosensitive transition was monitored by measuring the transition during heating and cooling cycles. The turbidity, microcalorimeter thermogram, and apparent size of the self-assembled particles of the aqueous mPEG-DC solution (1.0 wt.%) were measured. Turbidity (absorbance at 500 nm) was completely reversible during heating and cooling cycles (Fig. 8a). The microcalorimeter thermogram was reversible during heating and cooling cycles, except for a sharp peak at 32*–*33 °C (Fig. 8b). The small spike peak at the beginning of the endothermic band during the heating cycle was not observed in the cooling cycle. The endothermic band of 7.9 kcal/mol of polymer was measured during the heating cycle, whereas the exothermic band of 7.5 kcal/mol of polymer was measured during the cooling cycle. The size of the self-assembled micelles of mPEG-DC during the first and second heating cycles coincided, indicating reversibility of the self-assembly of mPEG-DC in water (Fig. 8c).

As a model drug, estradiol was used for drug delivery using the mPEG-DC thermosensitive micelles. Estradiol is a steroidal drug that has a similar structure to DC and is used as hormone therapy for women [31, 32]. The loading efficiency and loading content of the drug in the mPEG350-DC micelles were 92 ± 2% and 1.3 ± 0.3%, respectively. The micelle size determined by dynamic light scattering at 15 °C was practically the same; however, it slightly increased from 15 ± 6 to 16 ± 8 nm when the dug was loaded into the micelles, while the micelles maintained their spherical shapes (Supporting Information: Fig. S1). Estradiol is practically insoluble in water. The drug exhibited a solubility of 0.0039 mg/ml only when the Tween 80 concentration was 0.5 wt.%. To maintain the sink conditions for the drug during the in vitro drug release experiment, phosphate-buffered saline containing Tween 80 (4.0 wt.%) was used as release medium. The solubility of estradiol in the release medium was 0.33 mg/ml, indicating that approximately 90% of the loaded drug could be dissolved in the release medium (8.0 ml) (Supporting Information: Fig. S2).

**Fig. 9:**
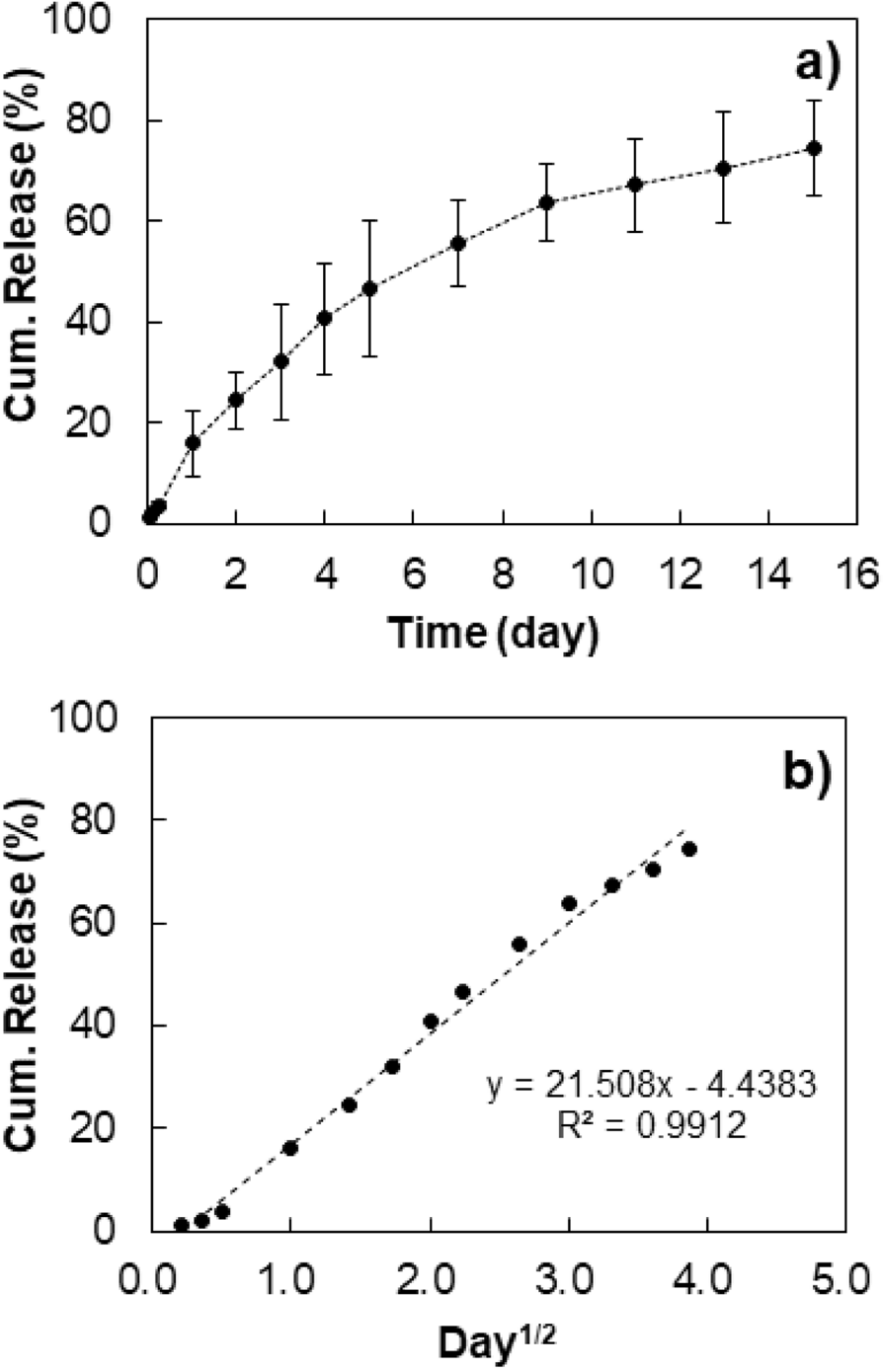
**a** Release profile of estradiol from thermosensitive micelles of mPEG350-DC. *n* = 3. **b** Replot of (**a**) against the square root of time

Considering in vivo applications, the drug release behavior was investigated at 37 °C. Estradiol was released from the thermosensitive mPEG-DC micelles over 16 days, with no significant initial burst (Fig. 9a). Because the estradiol-encapsulated micelles were coated with mPEG shells owing to the LCST behavior of the aqueous polymer solution, no initial burst was observed. The release profile showed a typical diffusion-based kinetics, as indicated by the proportionality to the square root of time (Fig. 9b). Typical micelle-based systems exhibit an initial burst release in the absence of LCST. A 45% release of docetaxel encapsulated in mPEG2000-docetaxel micelles was observed at 37 °C over 24 h [15]. Indomethacin encapsulated in poly(N-isopropylacrylamide)-cholic acid micelles showed a release of over 95% at 25 °C over 24 h; however, its release decreased to 45% at 37 °C over 24 h, as the surface coating of the micelles through the LCST behavior considerably reduced the initial burst release of the drug [33]. However, poly(N-isopropylacrylamide) poses toxicity concerns associated with residual monomers and the degradation products of isopropyl amine [34, 35]. The unique feature of the temperature-sensitive micelles with rigid cores is the absence of initial burst release above their LCST. In particular, our mPEG-DC is a promising thermosensitive micelles for biomedical applications, owing to the biocompatibility of both mPEG and DC.

## Discussion

Thermosensitive micelles were prepared using deoxycholic acid as a rigid core and mPEG as a shell. The LCST of the aqueous mPEG-DC solution was independent of concentration in a range of 0.1-10.0 wt.% and was observed at 30 − 35 °C. The transition temperature could be controlled by varying the PEG molecular weight and was 55-60 °C and 70-75 °C for mPEG-DC with 550 and 750 Da mPEG, respectively (Supporting Information: Fig. S3). Considering the potential for biomedical applications, this research focused on mPEG-DC with 350 Da mPEG. The rigidity of the DC core in the micelles was confirmed by ^1^H-NMR spectra in D_2_O as a function of temperature. In contrast with the flexible cores of PLGA, PTMC, and PPG, the DC core remains as a collapsed peak at above LCST, indicating the rigidity of the DC core of the mPEG-DC micelles. The unique micelles with rigid DC cores result in micelles with high reproducibility and reversibility in the thermal transition during heating and cooling cycles. In addition, estradiol encapsulated in the micelle exhibits release in a diffusion-controlled manner without an initial burst release. Previous studies reported the formation of mPEG-DC with PEG molecular weights of 1000 and 5000 Da [17]. Our mPEG350-DC is different from that reported in the previous works because it exhibits a LCST in the physiologically important temperature range of 30–40 °C. The CMC values of mPEG1000-DC and mPEG5000-DC were 0.43, and 0.36 wt.%, respectively, at 25 °C; these values are much higher than that of our thermosensitive mPEG350-DC (0.05 wt.%). The CMC value of mPEG2000-docetaxel was reported to be 0.088 wt.% [15]. A spherical-to-worm-like micelle transition was also reported to occur upon adding a surfactant (C_12_EG_3_ or C_22_EG_6_) with a flexible core to the mPEG-DC aqueous solution [36, 37]. The main difference between our study and these previous studies is that the latter focuses on nanostructural changes in binary mixtures, rather than on biomedical applications using thermosensitive micelles for drug delivery. In particular, our study demonstrated that thermosensitive micelles with rigid cores can be prepared by conjugating deoxycholic acid to small-molecular-weight (350 Da) PEG.

To conclude, in this paper we described a new thermosensitive rigid-core micelle with an LCST of 30 − 35 °C. The micelle exhibited thermoreversible behavior; the results demonstrated its potential significance as a drug delivery carrier. An in vivo pharmacokinetic study will be carried out in the future to further prove the practical efficacy of the thermosensitive mPEG350-DC micelle.

## Conclusions

In this paper, thermosensitive micelles with rigid cores were prepared using deoxycholic acid conjugated to low molecular weight mPEG. When 350 Da mPEG was conjugated to DC, it formed thermosensitive micelles with an LCST in the biologically important temperature range of 30 − 35 °C. The unique solution behavior of mPEG-DC was investigated by various instrumental methods. The core (DC)-shell (mPEG) structure was confirmed by hydrophobic dye solubilization, ^1^H-NMR in CDCl_3_ and D_2_O, TEM images, and dynamic light scattering. The temperature-dependent behavior was monitored by turbidity or LCST measurements, microcalorimetry, and dynamic light scattering. The endothermic peak of the LCST transition suggests that the transition is an entropy-driven process that is dominated by dehydration of the polymers. The liberated water molecules govern the entropy gain of the transition. The core rigidity of the mPEG-DC micelles was demonstrated using temperature-dependent ^1^H-NMR spectra of the polymer in D_2_O. The micelles exhibited a very thermoreversible transition and recovered their sizes through a cooling and heating cycle. The micelles encapsulated estradiol in their cores and is released it over 16 days in a diffusion-controlled manner.

## Supplementary Information

**Additional file 1: Fig. S1.** Size and morphology of mPEG350-DC micelles before and after estradiol loading at room temperature (15 °C). a) Micelle size distribution obtained using dynamic light scattering. b) TEM images of the mPEG350-DC micelles before and after estradiol loading. The scale bar is 50 nm. **Fig. S2.** Solubility of estradiol in phosphate buffered saline as a function of Tween 80 concentration. **Fig. S3.** LCSTs of aqueous mPEG-DC solution (1.0 wt.%) as a function of mPEG molecular weight. mPEG with 350, 550, and 750 Da were conjugated to DC to prepare mPEG350-DC, mPEG550-DC, and mPEG750-DC. LCST of the polymers are 30 − 35, 55 − 60, and 70 − 75 °C, respectively.

## Data Availability

For data requests, please contact the authors.
