# Supplementary material for: Thermosensitive core-rigid micelles of monomethoxy poly(ethylene glycol)-deoxy cholic acid
Source: Biomater Res. 2022 Apr 28;26:16. doi: 10.1186/s40824-022-00263-9 (PMC9052506; doi:10.1186/s40824-022-00263-9)

**Supplementary Information**

Thermosensitive core-rigid micelles of monomethoxy poly(ethylene glycol)-deoxy cholic acid

Jin Ok Han, Hyun Jung Lee, and Byeongmoon Jeong*

*Department of Chemistry and Nanoscience, Ewha Womans University, 52 Ewhayeodae-gil, Seodaemun-gu, Seoul, Korea*

* Corresponding author.

E-mail addresses: [bjeong@ewha.ac.kr](mailto:bjeong@ewha.ac.kr), Tel.: +82 2 3277 3411; Fax: +82 2 3277 3419

**Fig. S1** Size and morphology of mPEG350-DC micelles before and after estradiol loading at room temperature (15 ^o^C). a) Micelle size distribution obtained using dynamic light scattering. b) TEM images of the mPEG350-DC micelles before and after estradiol loading. The scale bar is 50 nm.


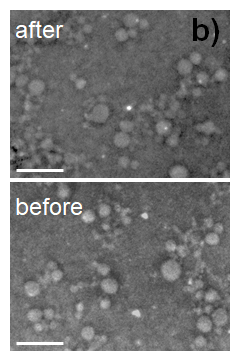

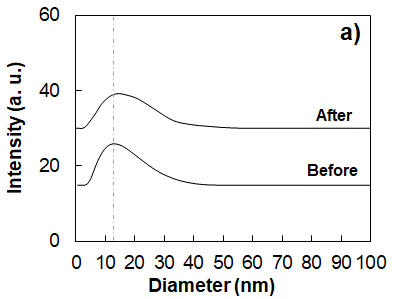


**Fig. S2** Solubility of estradiol in phosphate buffered saline as a function of Tween 80 concentration.


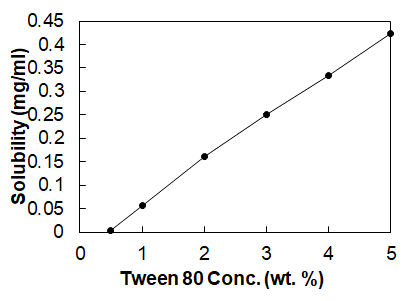


**Fig. S3** LCSTs of aqueous mPEG-DC solution (1.0 wt.%) as a function of mPEG molecular weight. mPEG with 350, 550, and 750 Da were conjugated to DC to prepare mPEG350-DC, mPEG550-DC, and mPEG750-DC. LCST of the polymers are 30−35, 55−60, and 70−75 ^o^C, respectively.


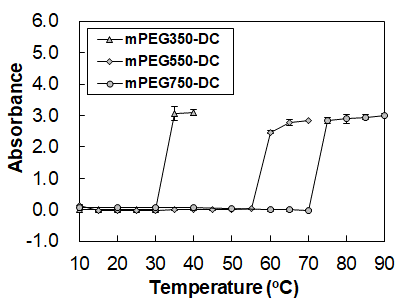

Supplement: Supplementary file 1 — Additional file 1: Fig. S1. Size and morphology of mPEG350-DC micelles before and after estradiol loading at room temperature (15 °C). a) Micelle size distribution obtained using dynamic light scattering. b) TEM images of the mPEG350-DC micelles before and after estradiol loading. The scale bar is 50 nm. Fig. S2. Solubility of estradiol in phosphate buffered saline as a function of Tween 80 concentration. Fig. S3. LCSTs of aqueous mPEG-DC solution (1.0 wt.%) as a function of mPEG molecular weight. mPEG with 350, 550, and 750 Da were conjugated to DC to prepare mPEG350-DC, mPEG550-DC, and mPEG750-DC. LCST of the polymers are 30 − 35, 55 − 60, and 70 − 75 °C, respectively. [file 40824_2022_263_MOESM1_ESM.doc]
